# Risks and Implications of Multiple Actionable Pathogenic Germline Variants Discovered by Panel-Based Cancer Predisposition Testing

**DOI:** 10.1200/PO-24-00951

**Published:** 2025-10-16

**Authors:** Catherine Neumann, Demitrios Dedousis, Michael J. Hall

**Affiliations:** ^1^ Department of Clinical Genetics, Fox Chase Cancer Center, Philadelphia, PA; ^2^ Department of Medical Oncology, Fox Chase Cancer Center, Philadelphia, PA

## Abstract

**PURPOSE:**

Growing use of multigene panels (MGPs) is increasing the number of patients identified with multiple pathogenic germline variants (PGVs) in cancer predisposition genes. This study characterizes the landscape of patients with multiple PGVs and identifies clinical settings where multiple PGVs affect management.

**MATERIALS AND METHODS:**

This is a single-institution retrospective cohort analysis comprising patients seen in the Department of Clinical Genetics and consented to the Risk Assessment Program (RAP) Registry who were evaluated with a MGP and found to have multiple PGVs.

**RESULTS:**

All patients tested between January 1, 2014, and January 1, 2024, and found to have multiple PGV are included. Sixty-four patients (64/7,961, 0.8%) from 58 families carried multiple PGVs, 22/64 (34%) patients carried at least two PGVs in high- or moderate-risk genes, and 33/64 (52%) carried at least two PGVs that result in potential management changes. Five percent (30/557) of all patients with a PGV in *BRCA1* or *BRCA2* also carried an additional PGV, while 7% (19/284) of patients with a PGV in a mismatch repair (MMR) gene also carried an additional PGV. Ten patients from nine unrelated families had both a PGV in *BRCA1* or *BRCA2* as well as a PGV in an MMR gene.

**CONCLUSION:**

Although the overall percentage of patients undergoing clinical genetic testing with multiple PGVs is small, a significant fraction of these patients could benefit from medical management changes because of the identification of multiple PGVs.

## INTRODUCTION

Because of the decreasing cost, increasing accessibility, and higher likelihood of identifying a germline pathogenic variant (PGV), the use of multigene panels (MGPs) is superseding single-gene testing or single-site/familial variant testing in the evaluation for hereditary cancer predispositions syndromes.^1-8^ Increased uptake of steadily larger MGPs in clinical practice will increase the number of patients identified with multiple cancer-predisposing PGVs.^1-5,7^ Despite this, there is currently limited information on patients with multiple PGV. The current literature reports that co-occurrence of multiple variants among populations tested with MGPs is rare (approximately 0.3%-3%)^2,5,9-12^ and that approximately 1%-4% of those who tested *positive* also had a second PGV.^1,5,7,8^ Patients are typically affected with malignancies associated with their individual PGVs^9^; however, unusual malignancies have been reported.^12,13^ Notably because of a lack of data, the clinical impact of multiple PGVs on a patient's cancer phenotype is unknown, and the screening and management approach for these patients and families must be customized on the basis of clinical experience or must follow guidelines for the individual PGVs.^10,14^

**CONTEXT**

**Key Objective**
How might identification of multiple pathogenic germline variants (PGVs) in cancer-predisposing genes in a high-risk patient change risk and alter clinical management?
**Knowledge Generated**
In our single-institution cohort, multiple PGVs occurred in 0.8% of patientswho had multiple gene panel testing. Thirty-four percent of those with multiple PGVs reported Ashkenazi Jewish ancestry, a population known to have undergone a founder effect. Fifty-nine percent of patients with multiple PGVs had at least one variant in either *BRCA1*, *BRCA2*, or one of the MMR genes associated with Lynch syndrome. We found that 52% of all patients with multiple PGVs could qualify for a change in surveillance, risk-reducing surgery, or antineoplastic therapy on the basis of their multiple variants according to National Comprehensive Cancer Network guidelines.
**Relevance**
Multigene panel testing should be recommended over single site or gene testing as the identification of multiple PGVs can change clinical management. This may be especially relevant in populations that have undergone a founder effect.

Unfortunately, despite the availability of MGP testing for hereditary cancer, many patients with a known familial PGV continue to undergo single-site genetic testing and therefore will not benefit from management recommendations associated with missed PGVs. In this study, we present the characteristics of all patients identified through the Fox Chase Cancer Center (FCCC) Risk Assessment Program (RAP) over a 10-year period who carry more than one PGV. The purpose of this study is to further characterize the landscape of patients found to carry multiple PGVs and to present exemplary patient cases where the identification of multiple PGVs affected management as well as the management lessons drawn from these cases.

## MATERIALS AND METHODS

This is a single-institution retrospective cohort analysis and chart review comprising patients seen in the FCCC RAP. Patients included in this analysis were referred to the Department of Clinical Genetics for cancer risk assessment on the basis of personal or family history suspicious for a hereditary cancer predisposition syndrome. All patients referred for genetic counseling undergo a detailed clinical intake and are asked to complete a health history questionnaire. After completing counseling and testing, patients are invited to join the prospective RAP registry. The registry currently includes over 14,000 patients accrued since 1999.

In this study, the RAP Registry was queried to identify patients evaluated with MGP between January 1, 2014, and January 1, 2024, and found to carry more than one PGV in one or more cancer predisposition genes. A variety of commercially available MGPs from multiple vendors ranging in size from 2 to 85 genes are routinely used by genetic counselors at FCCC. A small percentage of our retrospective study cohort includes patients with multiple PGVs who received MGP testing during the same period but elected not to join the RAP Registry. Genes and in some cases specific PGVs were categorized as either high-, moderate-, low-, or recessive-risk genes (Table 1). There is no standard definition of a high-penetrance gene versus a moderate-penetrance gene; however, we defined a moderate-penetrance gene/PGV as a relative risk of any cancer of 2-4 times that in the general population with high penetrance and low penetrance falling above and below that threshold, respectively.^15^ The potential for multiple PGVs to change management was determined on the basis of the most recent National Comprehensive Cancer Network guidelines^16,17^ and was agnostic of patient demographics. Management-changing PGVs included all high- and moderate-risk PGVs and *APC*^16^ I1307K but excluded all other low-risk PGVs (*CHEK2* [I157T], *CHEK2* [S428F],^18^ and *SPINK1* [N34S]) and all recessive-risk PGVs (*NBN*, *CFTR*, *FH* [K477dup], *NTHL1*, *MUTYH*, *WRN*, and *RECQL4*; Table 1). Patients with at least two management-changing PGVs were considered to have potential management changes (unless the management-changing PGVs occurred in the same gene). Patients with biallelic PGVs for autosomal recessive (AR) disorders such as *MUTYH* were excluded from this analysis. Sixty-four patients from 58 families were identified. Proband was defined as the first person in a family to establish care in the FCCC RAP. For the 64 patients with multiple PGVs, we confirmed through a chart review that probands were the first in their family to have a PGV identified and that nonprobands, that is, relatives, had a family member who had previously tested positive for at least one PGV. Clinical information on these patients, including review of pedigrees and MGP testing reports, was collected from the RAP registry and the electronic medical record by RAP personnel and the investigators. The resulting data set was stored in the REDCap (Nashville, TN)^19,20^ data management tool with all data stored on the servers managed by the Temple Health Information Systems Information Technology Department in a secure data center at all times. The RAP prospective registry is institutional review board (IRB)–approved, and the FCCC IRB reviewed this subprotocol (IRB 24-9013) and found it to be exempt. Descriptive statistics, performed by the investigators, were used to analyze the generated data set.

**TABLE 1.** Risk Category of Genes and Variants

Risk Category	Relative Risk	Genes
High-risk genes	>4	***BRCA1***, ***BRCA2***, ***PALB2***, ***MLH1***, ***MSH2***, ***MSH6***, ***PMS2***, ***FH***, ***APC***, ***CDKN2A***
Moderate-risk genes	2-4	***ATM***, ***BARD1***, ***BRIP1***, ***CHEK2***, ***RAD51D***
Low-risk genes/variants	<2	***APC (I1307K)***, *CHEK2 (I157T)*, *CHEK2 (S428F)*, *SPINK1 (N34S)*
Recessive-risk genes/variants	NA	*NBN*, *CFTR*, *FH (K477dup)*, *NTHL1*, *MUTYH*, *WRN*, *RECQL4*

NOTE. Bold indicates management-changing variants.

Abbreviation: NA, not applicable.

## RESULTS

**FIG 1.**
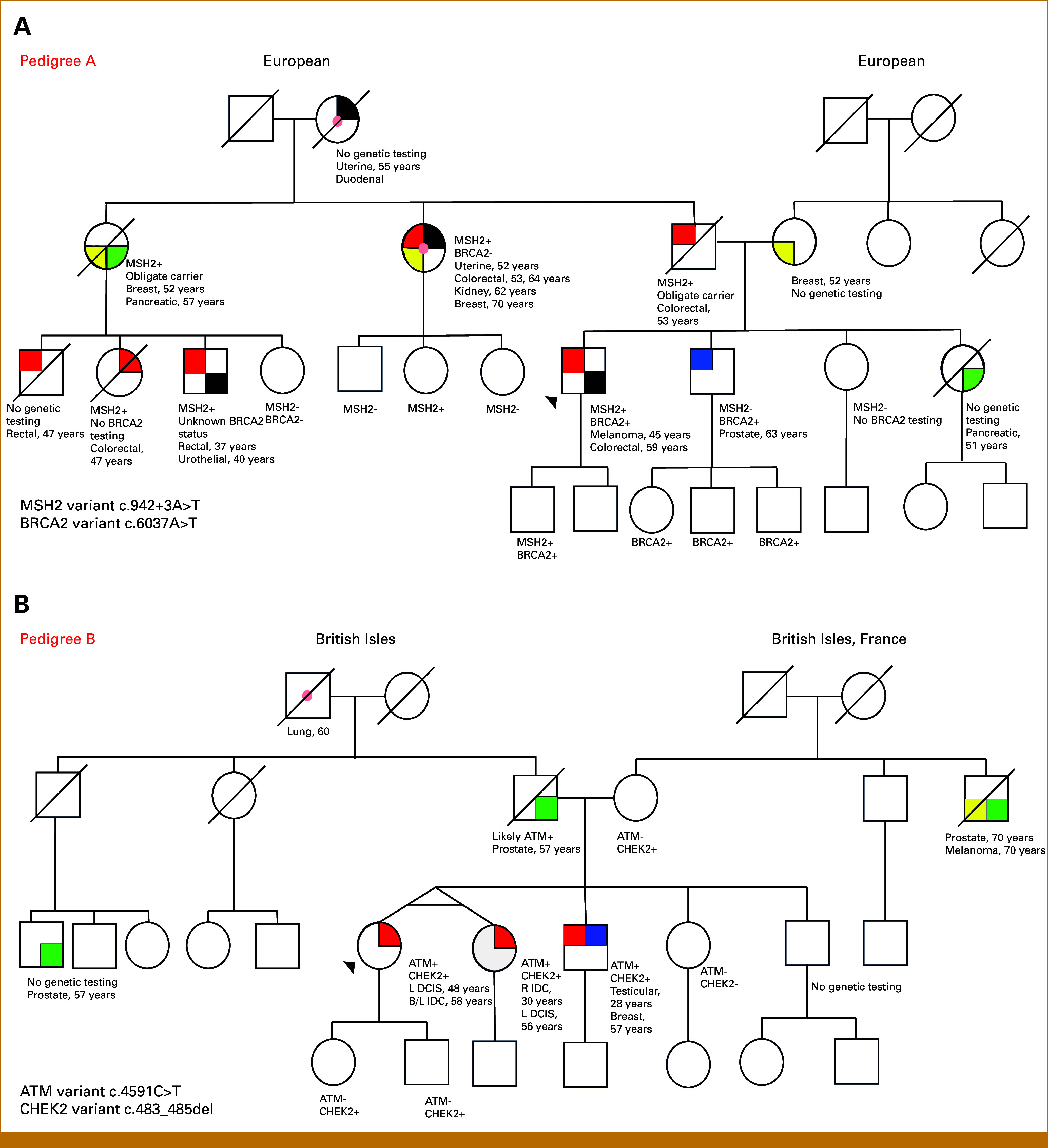
Pedigrees of selected families carrying two high/moderate penetrance variants. Pedigree A highlights a family with *BRCA2* and *MSH2* PGVs. Testing performed on extended paternal relatives confirmed the *MSH2* PGV was paternally inherited. Familial testing has not yet determined the parental origin of the *BRCA2* PGV. Pedigree B highlights a family with *ATM* and *CHEK2* PGVs first identified in identical twin sisters. Testing performed on the twins' mother confirmed the *CHEK2* PGV was maternally inherited while the *ATM* PGV was likely paternally inherited. DCIS, ductal carcinoma in situ; IDC, invasive ductal carcinoma; PGV, pathogenic germline variant.

Seven thousand nine hundred sixty-one patients had MGP testing from January 1, 2014, to January 1, 2024. Of these, 6,106 (77%) were probands (first to establish care) in the FCCC RAP program, while 1,710 (21.5%) were relatives of probands. Proband status was not available for 145 (1.8%) patients. One thousand eight hundred eighteen (22.8%) patients tested with a MGP had a positive result. Over 10 years, 64 patients (64/7,961, 0.8%) tested positive for more than one PGV (Data Supplement, Table S1) and 58/64 (90.6%) of these patients were the first person in their family to test positive for multiple PGV. Forty-one/64 of these patients (64%) had no known family history of a PGV identified, while 23 (36%) patients had a relative with at least one known PGV. Overall, 0.7% (41/6,106) of probands tested and 1.3% (23/1,710) of relatives tested had multiple PGVs, and in total, 3.5% (64/1,818) of patients with any positive result on germline testing had multiple PGVs identified. Over half of the patients carrying multiple PGVs had a personal history of cancer (42/64, 66%) with an average age of onset of 53 years, and 8/42 (12.5%) had multiple primary cancers. In Pedigree A, the proband, and in Pedigree B, the proband's brother (Fig 1) serve as examples of patients with multiple PGVs that have multiple primary malignancies. The cohort with multiple PGVs was 98.4% non-Hispanic White with one third of patients (22/64, 34%) reporting Ashkenazi Jewish (AJ) ancestry (Table 2). As a comparison, in the overall RAP data, patients' ancestries were 10% AJ, 12% African American, 3% Asian, 0.18% Native American/Pacific Islander, 76% White/European, 4% Hispanic, and 9% other/not reported.

**TABLE 2.** Patient Characteristics

Characteristic	Frequency No. (%)
Sex	
Male	26 (40.6)
Female	38 (59.4)
Race	
White/Caucasian	63 (98.4)
African American/Black	1 (1.6)
Asian	0 (0)
Other	0 (0)
Ethnicity	
Hispanic	1(1.6)
Non-Hispanic	63 (98.4)
Ashkenazi Jewish ancestry	
Yes	22 (34.3)
No	42 (65.6)
Personal history of cancer	
Yes	42 (65.6)
No	22 (34.3)
Multiple primary cancers	
Yes	8 (12.5)
No	56 (87.5)
Proband	
Yes	41 (64)
No	23 (36)
Cancer type	
Breast	12 (18.7)
Ovarian	7 (10.9)
Endometrial	1 (1.6)
Colorectal	8 (12.5)
Prostate	8 (12.5)
Pancreatic	4 (6.2)
Other	9 (14.0)
Carrier of *BRCA1* or *BRCA2* pathogenic variant	
Yes	28 (43.7)
No	36 (56.3)
Carrier of *MLH1*, *MSH2*, *MSH6*, *PMS2*, or *EPCAM* pathogenic variant	
Yes	20 (31.2)
No	44 (68.8)

**FIG 2.**
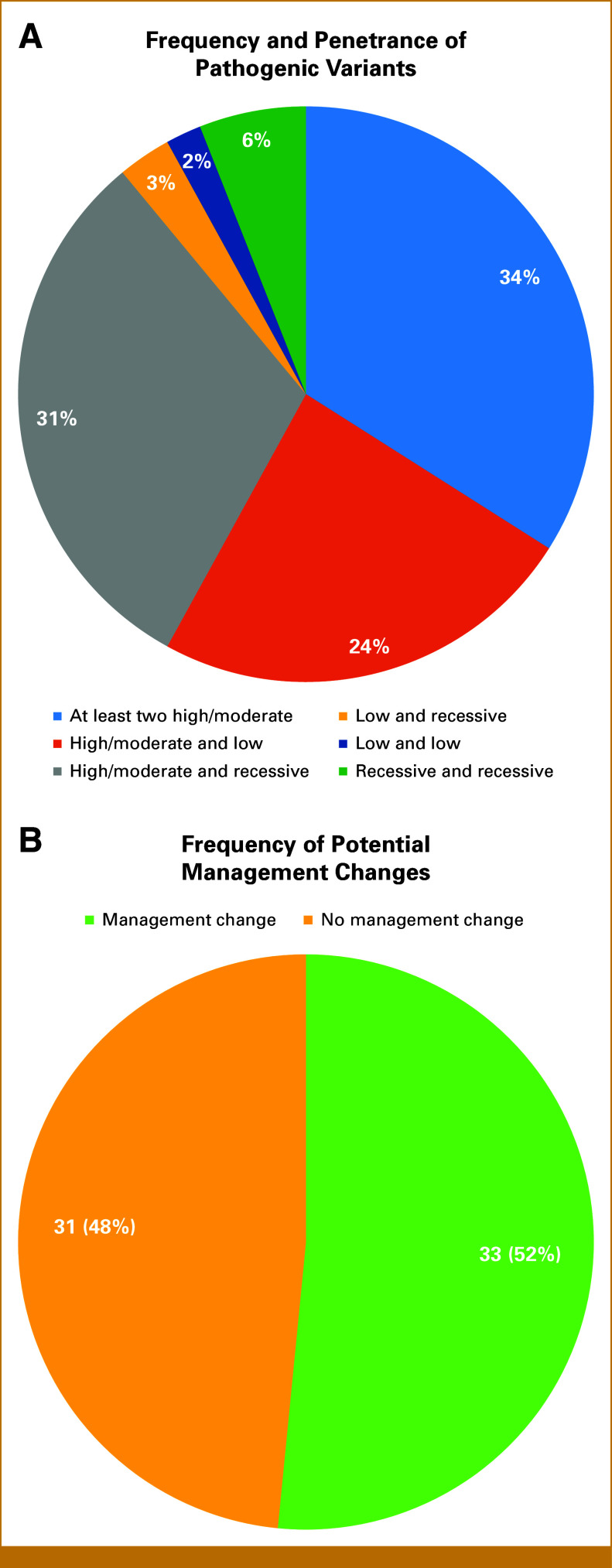
(A) Frequency of pathogenic variants of varying risk identified in this cohort. (B) Frequency of multiple pathogenic variants with potential management implications.

Autosomal dominant PGVs that were high penetrance, moderate penetrance, and low penetrance were identified, as were PGVs inherited in an AR fashion. Two or more PGVs in high-/moderate-risk genes were carried by 22/64 (34%) patients, while 15 others (24%) carried a PGV in a high-/moderate-risk gene and a low-risk gene. An additional 20 (31%) had a PGV in a high-/moderate-risk gene and a recessive-risk gene, four (6%) had two PGVs in recessive-risk genes, two (3%) had a PGV in both a low-risk gene and a recessive-risk gene, and one (2%) had two PGVs in low-risk genes (Fig 2A). Potential medical management changes were associated with recognition of a second PGV in a high-/moderate-risk gene. In our cohort, 33/64 (52%) PGV carriers were found to have a second PGV with implications of a potential change of medical management recommendations in terms of screening, risk reduction, or cancer-directed therapies (Fig 2B). Below, we highlight two families found to carry two high-/moderate-penetrance variants (Fig 1). Finally, 27/64 patients (42%) were found to be carriers of at least one recessive condition. The Data Supplement (Table S1) provides a list of all cohort patients with multiple PGVs plus their discrete variants and penetrance classifications.

Patients carrying multiple PGVs that include the *BRCA1*/*2* genes and/or the mismatch repair (MMR) genes are important to identify because of the prevalence, high cancer risks, and well-developed management guidelines associated with these genes. In our cohort, 38/64 (59%) patients from 36 families carried a PGV in the *BRCA1* or *BRCA2* gene and/or a MMR gene. This includes 10 patients from nine distinct families carrying both a *BRCA1* or *BRCA2* PGV as well as a PGV in an MMR gene. Five of these patients were the proband in their family. The proband in Pedigree A (Fig 1) is an example of one of these patients. Panel testing is particularly valuable to identify patients with multiple PGVs because of the high prevalence of PVs in the *BRCA1*/*2* and MMR genes, which lead to significant changes in screening and management for patients. Among patients who underwent MGP testing and were found to carry a *BRCA1* or *BRCA2* PGV, 5% (30/557) also carried at least one additional high-risk, moderate-risk, low-risk, or recessive-risk PGV. Similarly, 7% (19/284) of patients who underwent MGP testing and were found to carry a PGV in an MMR gene also carried at least one additional PGV.

## DISCUSSION

The frequency of patients in our study with multiple PGVs (0.8%) is in line with other studies that show a frequency of approximately 0.3%-3%.^2,5,9-12^ 3.5% of patients who tested positive on MGPs had multiple PGVs, a similar rate to previously published results (approximately 1%-4%).^1,5,7,8^ In our cohort, 36% of patients with multiple PGVs were identified because of cascade testing. Because these individuals had a higher pretest probability of carrying at least 1 PGV, the frequency of multiple PGVs may be inflated here compared with the general population. Notably, we found the rate of patients with multiple PGVs is higher (1.3% *v* 0.7%) among relatives of the proband, who are likely to have a familial PGV, compared with the probands themselves. In addition, those with multiple known familial variants may be more likely to seek out genetic testing than those with other indications. Finally, the probability of identifying multiple PGVs has clearly increased with the uptake of steadily larger MGPs. To this end, our study is limited by the heterogeneous nature of the MGPs used and that the MGPs offered changed over the 10-year period from which data were collected.

It is difficult to draw conclusions regarding the average age of cancer onset reported here (53 years) for carriers of multiple PGVs because our cohort spans multiple cancer types and multiple predisposition genes. Previous studies, specific to breast cancer risk, suggest that age of onset and risk for bilateral breast cancer are not increased in patients who carry multiple PGVs in breast cancer predisposition genes.^11,13^ In our study's cohort, those with multiple PGVs appear to have a more severe phenotype in terms of risk of developing multiple primary malignancies. Notably, our finding that 12.5% of patients with multiple PGVs reported multiple primary cancers is similar to the 20% reported in an earlier study.^5^

Although we found that the risk of identifying multiple PGVs in each patient is low, the chance that identifying multiple PGVs will change clinical management is significant (52%). Furthermore, most (59%) additional PGVs identified in the patients here were concentrated in genes causative for hereditary breast and ovarian cancer (HBOC) and Lynch syndrome (Table 2), diseases for which we have detailed management guidelines.^16,17^ Among our patients, there are concrete clinical implications to identifying these additional PGVs in terms of screening and risk reduction. For instance, the proband in Pedigree A (Fig 1) would benefit from both Lynch syndrome– and HBOC-directed screening. Patients in this family with an *MSH2* PGV and a PGV in *BRCA2*, depending on their age and sex, qualify for additional screening for breast, prostate, and pancreas cancer, and risk-reducing surgery for ovarian cancer.^17^ This is in addition to the screening and risk-reducing procedures already recommended for *MSH2* Lynch syndrome, which was detected earlier in this family.^16^ Beyond HBOC and Lynch syndrome, identification of additional low-penetrance PGVs can also in some cases change management. For instance, multiple guidelines advocate for a lower age of screening for colorectal cancer in patients with the low-penetrance *APC* I1307K variant.^16,21^ In our cohort, there were 13 patients where the identification of a second *APC* I1307K variant in addition to a PGV in a gene that does not predispose to GI malignancies altered management. Having two PGVs in moderate-penetrance genes can also have screening and management implications, as exemplified by the family in Pedigree B (Fig 1). Multiple individuals in this family including the proband and her identical twin sister and affected brother would qualify for pancreatic cancer screening with abdominal imaging at age 50 years because of their PGV in *ATM* in addition to the enhanced breast cancer screening recommended for females with either PGV in *ATM* or moderate-risk PGV in *CHEK2*.^17,22^

Beyond screening and prophylaxis, identification of additional PGVs can also affect selection of cancer-directed therapies. In those with PGVs in *BRCA1* or *BRCA2* or in some cases, other homologous recombination repair (HRR) genes such as *PALB2* (Data Supplement, Table S1) poly ADP ribose polymerase inhibitors (PARPi) can be used to treat solid tumors such as breast, pancreatic, prostate, and uterine cancers.^23-38^ In addition, platinum-based chemotherapy regimens are preferred in treatment of pancreatic cancer in patients with PGVs in *BRCA1*, *BRCA2*, or *PALB2*.^39^ Patients found to have Lynch syndrome are also likely to benefit from changes in their antineoplastic agents both as frontline systemic therapy or after other standard-of-care treatments are exhausted. The majority of Lynch syndrome–related cancers are MMR deficient (dMMR)^40^ and dMMR solid tumors are highly susceptible to immune checkpoint blockade.^41^ For example, immune checkpoint inhibitor (ICI) therapy is the preferred first-line treatment for patients with metastatic dMMR colorectal cancer. Patients treated with ICI had improved progression-free survival compared with chemotherapy.^42,43^ In our cohort, Pedigree A (Fig 1) is a prime example of a family where awareness of both the *BRCA2* and *MSH2* pathogenic variants can alter selection of cancer-directed therapeutics with PARPi becoming an option for HBOC-associated breast and prostate cancers and ICI becoming an option for dMMR colon and uterine cancers.^44-46^

The high likelihood of an additional PGV having specific changes in management suggests that all patients who qualify for cancer genetic testing should be offered a MGP over single-site or single-gene testing even when a familial variant is present. Patients undergoing single-site cascade testing are at risk of receiving inaccurate risk assessment on the basis of incomplete ascertainment of germline cancer risks. Even identification of carriers of AR conditions (Fig 2A) such as *MUTYH* polyposis can inform family planning and preconception carrier testing of reproductive partners.^47^ Carriers for AR conditions identified by cancer MGP testing are common with carriers of *MUTYH* polyposis alone, representing 1.5%-2.0% of the general population.^48,49^ However, insurance coverage for MGP when a known familial variant is present may be a barrier for some patients.^50,51^ MGP testing is especially important in patients with AJ ancestry and should be strongly considered in other populations known to have undergone a founder effect and therefore are more likely to have multiple PGVs.^48^ Those with AJ ancestry were overrepresented in this cohort with multiple PGVs compared with the overall RAP cohort (34% *v* 10%) as were those with White/European ancestry (98% *v* 76%). The overrepresentation of these populations is likely driven by founder variants common in these populations such as the *APC* I1307K^52^ and *BRCA1* c.5266dupC,^53^ PGVs that are known to be enriched in AJ individuals. These common founder variants are partially responsible for the prevalence of patients with multiple PGVs in this cohort. However, it is important to note, as illustrated by the two families (Fig 1) of non-AJ ancestry affected by multiple PGVs, that families from populations not traditionally associated with increased risk of hereditary cancer predispositions can also be affected by multiple PGVs and should be offered MGPs. It is well established that non-European ancestry populations are underrepresented in genetic databases,^54^ and it is likely that because of socioeconomic barriers, patients of non-European ancestry are underrepresented in the cohort we analyzed.^55,56^ The overrepresentation of AJ and White/European populations in this cohort for the above-stated reasons do limit the applicability of the data from this cohort to the general population.

Disclosing the results of MGP testing in those found to have multiple PGVs as well as crafting individualized surveillance plans is a challenge, given the limited data available on this patient population. We have multiple recommendations on the basis of our clinical experience with patients with multiple PGVs of best practices to improve care. A genetic counselor or provider with clinical genetics training should be involved in the results disclosure to provide accurate cancer risk assessment and recommend appropriate cascade testing. Additionally, it is useful to provide the patient with a results disclosure letter discussing all PGVs and outlining a single surveillance plan that the patient can reference. In complex cases, presentation to a multidisciplinary review meeting can allow providers to come to a consensus regarding the clinical relevance of all PGVs and appropriate surveillance plan. Given that many patients with multiple PGVs will require complex surveillance plans that include numerous visits and examinations with multiple screening modalities, we recommend referral to specialty centers with multidisciplinary cancer predisposition clinics. Multidisciplinary clinics serve as a centralized medical home for this population to reduce the burden of multiple visits and improve care coordination. This allows for changes in medical management to be appropriately disseminated to the patient and their providers as we learn more not only about the risk from individual variants but how multiple PGVs work in tandem to increase cancer risk.

In conclusion, this single-institution cohort retrospective analysis provides a descriptive analysis of patients found to carry multiple PGVs in genes associated with hereditary cancer risk. Although the overall percentage of patients with multiple PGVs identified in this cohort was small, a significant percentage of these patients' management recommendations could potentially change because of the identification of multiple PGVs. This suggests there is clinical utility to identifying multiple PGVs and supports use of MPGs even when there is a known familial variant. More research is needed to determine the optimal screening and management of those with multiple PGVs; however, in the absence of data, it is prudent to apply screening and management guidelines of both identified syndromes.
