# Supplementary material for: Risks and Implications of Multiple Actionable Pathogenic Germline Variants Discovered by Panel-Based Cancer Predisposition Testing
Source: JCO Precis Oncol. 2025 Oct 16;9:e2400951. doi: 10.1200/PO-24-00951 (PMC12537039; doi:10.1200/PO-24-00951)
Supplement: Supplementary file 1 [file po-9-e2400951-s001.pdf]

Supplemental Table 1

	Sex	Personal history of cancer	Ashkenazi Jewish Ancestry	Pathogenic Variants	Proband	Penetrance	Potential Management Change
1	Male	No	No	*BRCA2* c.2041delA<br>*NTHL1* c.268C>T	No	High penetrance and Recessive risk	No
2	Male	Prostate cancer at age 66	Yes	*BRCA1* c.66_67delAG<br>*APC* c.3920T>A (I1307K)	No	High penetrance and Low penetrance	Yes
3	Female	No	No	*BRCA1* c.5266dupC<br>*CHEK2* c.470T>C (I157T)	No	High penetrance and Low penetrance	No
4	Female	No	Yes	*BRCA2* c.5946delT<br>*CHEK2* c.1283C>T (S428F)<br>*APC* c.3920T>A (I1307K)	No	High penetrance, and Low penetrance, and Low penetrance	Yes
5	Female	No	Yes	*BRCA1* c.66_67delAG<br>*BRCA2* c.5946_5946delT	No	High penetrance and High penetrance	Yes
6	Female	Colon cancer at age 70	No	*BRCA2* c.5350_5351delAA<br>*MSH6* c.694C>T	No	High penetrance and High penetrance	Yes
7	Female	No	Yes	*BRCA2* c.5946_5946delT<br>*PMS2* c.943C>T	Yes	High penetrance and High penetrance	Yes
8	Male	Colon at age 58	No	*BRCA2* c.6037A>T<br>*MSH2* c.942+3A>T	No	High penetrance and High penetrance	Yes

9	Male	No	No	*BRCA2* c.6037A>T<br>*MSH2* c.942+3A>T	No	High penetrance and High penetrance	Yes
10	Female	Ovarian cancer at 62	No	*BRCA1* c.3756_3759delGTCT<br>*PMS2* c.2095G>C	Yes	High penetrance and High penetrance	Yes
11	Female	No	No	*BRCA1* c.5071_5072insA<br>*PMS2* c.3G>A	Yes	High penetrance and High penetrance	Yes
12	Female	Bilateral breast cancer at 41, Endometrial cancer at 43, Colon cancer at 55, Sebaceous carcinoma at 68	No	*BRCA2* c.2833_2834insTT<br>*MSH6* c.3656C>A	Yes	High penetrance and High penetrance	Yes
13	Male	Prostate cancer at age 57, Sebaceous carcinoma at age 62	No	*BRCA1* c.5266dupC<br>*MSH2* c.1276+1G>A	Yes	High penetrance and High penetrance	Yes
14	Female	DCIS at age 38	Yes	*BRCA2* c.5946delT<br>*PMS2* dup exons 11-12	No	High penetrance and High penetrance	Yes
15	Male	Sarcoma at age 57	No	*BRCA1* c.5096G>A<br>*MSH6* c.578del	No	High penetrance and High penetrance	Yes
16	Female	Ovarian cancer at age 49	No	*BRCA1* c.4158_4162del<br>*MUTYH* c.536A>G	No	High penetrance and Recessive risk	No

17	Male	Prostate cancer at age 60	No	*BRCA2* c.2808_2811del *NTHL1* c.268C>T	Yes	High penetrance and Recessive risk	No
18	Female	Ovarian cancer at age 62	No	*BRCA2* c.8487+1G>A *NTHL1* c.268C>T	Yes	High penetrance and Recessive risk	No
19	Male	Rectal cancer at 49	Yes	*BRCA2* c.5576_5579delTTAA *APC* c.3920T>A (I1307K)	Yes	High penetrance and Low penetrance	Yes
20	Male	No	Yes	*BRCA2* c.5576_5579delTTAA *APC* c.3920T>A (I1307K)	No	High penetrance and Low penetrance	Yes
21	Female	No	Yes	*BRCA1* c.66_67delAG *APC* c.3920T>A (I1307K)	Yes	High penetrance and Low penetrance	Yes
22	Female	Ovarian cancer at 51	Yes	*BRCA1* c.66_67delAG *APC* c.3920T>A (I1307K)	Yes	High penetrance and Low penetrance	Yes
23	Male	Prostate cancer at age 73 and Breast cancer at age 74	Yes	*BRCA2* c.5946_5946delT *APC* c.3920T>A (I1307K)	Yes	High penetrance and Low penetrance	Yes
24	Female	No	No	*BRCA2* c.5350_5351delAA *PALB2* c.3113G>A	Yes	High penetrance and High penetrance	Yes
25	Female	Bladder cancer at age 38	No	*BRCA1* c.3756_3759delGTCT *CHEK2* c.1100delC	Yes	High penetrance and Moderate penetrance	Yes
26	Male	Prostate cancer at age 51	No	*BRCA1* c.4799dup *CHEK2* c.444+1G>A	Yes	High penetrance and Moderate penetrance	Yes
27	Male	Bladder cancer at age 56, Prostate cancer at 56,	No	*BRCA2* c.1800T>G *NBN* c.2099del	Yes	High penetrance and Recessive risk	No

		Papillary RCC at 62					
28	Male	Prostate cancer at age 65	No	*BRCA2* c.1813_1813delA<br>*FH* c.1431_1433dupAAA	Yes	High penetrance and Recessive risk	No
29	Female	No	No	*MSH6* c.3647-1G>A<br>*APC* c.3920T>A (I1307K)	No	High penetrance and Low penetrance	Yes
30	Male	No	No	*MLH1* c.350C>T<br>*MUTYH* c.1187G>A	No	High penetrance and Recessive risk	No
31	Female	No	Yes	*PMS2* c.137G>T<br>*MUTYH* c.494A>G	Yes	High penetrance and Recessive risk	No
32	Female	Ovarian cancer at 74	No	*PMS2* c.137G>T<br>*MUTYH* c.734G>A	Yes	High penetrance and Recessive risk	No
33	Male	No	No	*PMS2* c.861_864del<br>*MUTYH* c.536A>G	Yes	High penetrance and Recessive risk	No
34	Male	Rectal cancer at 33	No	*MSH2* c.187delG<br>*MUTYH* c.455G>A	Yes	High penetrance and Recessive risk	No
35	Male	Colon cancer at age 44	Yes	*MSH2* c.1906G>C<br>*APC* c.3920T>A (I1307K)	No	High penetrance and Low penetrance	Yes
36	Female	Ovarian cancer at age 44	No	*MSH6* c.3261dupC<br>*RAD51D* c.1A>G	Yes	High penetrance and Moderate penetrance	Yes
37	Male	Pancreatic cancer at 54	No	*MSH6* c.3226C>T<br>*BRIP1* c.2992_2993delAA	Yes	High penetrance and Moderate penetrance	Yes
38	Male	No	No	*PMS2* del exons 6-7<br>*NBN* c.1903A>T	No	High penetrance and Recessive risk	No

39	Male	Pancreatic cancer at 65	No	*MUTYH* c.1187G>A<br>*NBN* c.657_661delACAAA	Yes	Recessive risk and Recessive risk	No
40	Female	No	No	*BRIP1* c.2492+2dup<br>*FH* deletion (entire coding sequence)<br>*MUTYH* c.1187G>A	Yes	Moderate penetrance and High penetrance and Recessive risk	Yes
41	Female	Breast cancer X2 at 44; Colon cancer at 57; Pancreatic cancer at 67	No	*CHEK2* c.1100delC<br>*CHEK2* c.190G>A<br>*MUTYH* c.1187G>A	Yes	Moderate penetrance and Moderate penetrance and Recessive risk	No
42	Female	Colorectal cancer at 48	No	*BRIP1* c.2684_2687del<br>*MUTYH* c.536A>G	Yes	Moderate penetrance and Recessive risk	No
43	Female	No	No	*RAD51D* c.649G>T<br>*RAD51D* c.655C>T<br>*In cis	No	Moderate penetrance and Moderate penetrance	No
44	Female	L DCIS at 37; R breast cancer at 61	No	*CHEK2* c.277delT<br>*CHEK2* c.1420C>T<br>*In trans	Yes	Moderate penetrance and Moderate penetrance	No
45	Female	Colorectal cancer at 27	Yes	*APC* c.994C>T<br>*NTHL1* c.268C>T	Yes	High penetrance and Recessive risk	No
46	Male	Prostate cancer at 50	No	*ATM* c.2921+1G>A<br>*APC* c.3920T>A (I1307K)	No	Moderate penetrance and Low penetrance	Yes
47	Male	No	No	*ATM* c.2921+1G>A<br>*APC* c.3920T>A (I1307K)	No	Moderate penetrance and Low penetrance	Yes
48	Female	No	No	*ATM* c.2921+1G>A<br>*APC* c.3920T>A (I1307K)	No	Moderate penetrance and Low penetrance	Yes
49	Female	Breast cancer at 72	Yes	*ATM* c.1027_1030del<br>*APC* c.3920T>A (I1307K)	No	Moderate penetrance and Low penetrance	Yes

50	Female	Breast cancer at 43	Yes	*ATM* c.1027_1030del<br>*APC* c.3920T>A (I1307K)	Yes	Moderate penetrance and Low penetrance	Yes
51	Female	Breast cancer at 43	No	*ATM* c.7380del<br>*BARD1* c.2001+1G>A	Yes	Moderate penetrance and Moderate penetrance	Yes
52	Female	R DCIS at 48, Bilateral breast cancer at 58	No	*ATM* c.4591C>T<br>*CHEK2* c.483_485delAGA	No	Moderate penetrance and Moderate penetrance	Yes
53	Female	R Breast cancer at 30; L Breast cancer at 56	No	*ATM* c.4591C>T<br>*CHEK2* c.483_485delAGA	Yes	Moderate penetrance and Moderate penetrance	Yes
54	Male	Pancreatic cancer at 58	No	*ATM* c.496+5G>A<br>*CFTR* TG11-5T	Yes	Moderate penetrance and Recessive risk	No
55	Female	Breast cancer at 55	No	*ATM* c.3G>A<br>*CFTR* c.1521_1523delCTT	Yes	Moderate penetrance and Recessive risk	No
56	Male	Kidney cancer at 49	Yes	*CHEK2* c.1283C>T<br>*CFTR* TG11-5T	Yes	Low penetrance and Recessive risk	No
57	Male	Pancreatic cancer at 61	No	*CDKN2A* c.301G>T<br>*CFTR* c.1521_1523delCTT	Yes	High penetrance and Recessive risk	No
58	Female	Breast cancer at 56	Yes	*APC* c.3920T>A (I1307K)<br>*CHEK2* c.1283C>T (S428F)	Yes	Low penetrance and Low penetrance	No
59	Female	No	Yes	*NTHL1* c.859C>T<br>*WRN* c.3030_3033del	No	Recessive risk and Recessive risk	No
60	Female	Melanoma at 60; Colorectal cancer at 61	No	*RECQL4* c.1568_1573delinsCCCCC<br>*CFTR* TG11-5T	Yes	Recessive risk and Recessive risk	No
61	Female	Ovarian cancer at 61	No	*NBN* c.657661delACAAA<br>*NTHL1* c.550-1G>T	Yes	Recessive risk and Recessive risk	No

62	Male	Melanoma at 72	Yes	*CHEK2* c.444+1G>A<br>*NBN* c.657_661delACAAA	Yes	Moderate penetrance and Recessive risk	No
63	Female	No	No	*SPINK1* c.101A>G<br>*WRN* c.3044G>A	Yes	Low penetrance and Recessive risk	No
64	Male	Kidney cancer at 52	Yes	*CHEK2* c.1283C>T (S428F)<br>*NBN* c.1903A>T	Yes	Low penetrance and Recessive risk	No
